# Supplementary material for: Prevention of congenital toxoplasmosis in France using prenatal screening: A decision-analytic economic model
Source: PLoS One. 2022 Nov 4;17(11):e0273781. doi: 10.1371/journal.pone.0273781 (PMC9635746; doi:10.1371/journal.pone.0273781)
Supplement: S1 File — (DOCX) [file pone.0273781.s001.docx]

What follows explains derivations of both treatment and accommodation costs based on levels of disability and of productivity losses that are presented in Table 3. When sources for costs in France were not available, we used costs in Germany (adjusting for differences in consumer prices between the two countries) as a proxy for French costs since the two countries have similar health systems and economies. The German data come from Table 7.9 page 134 in Walter et al.1 Those data are used to estimate schooling costs for children with disabilities and the earnings loss of caregivers.

Cost data were adjusted for consumer price inflation between the time the data were collected and January 2020. Consumer prices were adjusted using the harmonized index of consumer prices (HICP) produced by the European Central Bank.^2^ The HICP is the official measure of consumer price inflation in the EU. In general, price indices are constructed by referencing changes in prices of the same fixed basket of goods and services. The HICP uses the same basket in all European countries, hence its label “harmonized.”

**Treatment costs for mild cognitive impairment (CognitiveMild)** The costs of child and adolescent disorders of the brain in France are used as a proxy for the cost of mild cognitive disorders. The annual cost of child and adolescent disorders is found in Table 11, page 734 of Gustavsson *et al*,^3^ which gives annual cost per person in France as weighted means for all diagnoses and age groups within the disorder. Gustavsson *et al.*’s figures cover several developmental disorders and include direct and indirect costs. Indirect costs for this category include parent earnings loss. Gustavsson *et al.* estimate the annual cost of child and adolescent disorders in France in 2010 to be €3833. We adjust that figure for consumer price inflation from 2010 to 2020 in France and find the annual cost of child and adolescent disorders to be €4,276 in France in 2020.^2^ Summing over 18 years and discounting 3% annually, we estimate the lifetime costs of mild cognitive impairment in France to be €54,124.

This is a conservative approach that likely understates the true cost: we base our cost estimate on the costs of child and adolescent disorders, so our calculations go from infancy to age 18. Nevertheless, it is likely that the psychological and economic burdens of these disorders continue into the person’s adulthood.

**Treatment costs for severe cognitive impairment (CognitiveSevere)** The costs of mental retardation were used as a proxy for the costs of severe cognitive disorders. The annual costs of mental retardation in France are found in Table 11, page 734 of Gustavsson *et al.*^3^ Their estimate includes direct and some indirect costs but does not include lifetime earnings loss for the parent or for the child. Gustavsson *et al*. estimate an annual cost of €11,522 in France in 2010. We adjust that figure for consumer price inflation from 2010 to 2020 in France and find the annual cost of mental retardation in 2020 to be €11,522 in 2020 consumer prices.^2^ Life expectancy in France is 82.8 years.^4^ Summing over 82.8 years beginning at age 0 and discounting 3% annually, we estimate the lifetime costs of severe cognitive impairment in France to be €406,634.

**Earnings loss from severe cognitive impairment of child (ChildEarnLoss)** is the life-time earnings loss due to severe impairment that prevents gainful employment. The Organisation of European Cooperation and Development (OECD) reports the mean French annual wage for full-time, full-year employees for 2020 to be €39,099.^5^ The normal retirement age in France is 62 years. Summing over years 18 to 62 and discounting 3% annually, we estimate the wages lost from severe cognitive impairment to be €564,858.

**Special education costs for children with mild cognitive impairment (SpecEdCognitiveMild) is** based on Table 7.9, page 134. in Walter *et al*.^1^ They estimate annual costs of schooling in Germany in 2008 for those with mild cognitive impairment to be €6,126. Adjusting German schooling costs in 2008 to French consumer prices in 2020, we find the 2020 annual cost of schooling in France to be €7,031. The children receive 15 years of special schooling from age 4 to 18. Summing costs of schooling for children with mild cognitive impairment for 10 years beginning at age 4 and discounting 3% annually, we estimate the costs of their schooling to be €74,967.

**Special education costs for children with severe cognitive impairment (SpecEdSevereCog)** is based on the Table 7.9, page 134 in Walter *et al*.^1^ They estimate the cost of schooling for children with severe cognitive impairment in Germany in 2008 to be €44,835 per year for 20 years. Adjusting German schooling costs in 2008 to French consumer prices in 2020, we find the 2020 annual cost of schooling for those with severe cognitive impairment in France to be €51,728.^2^ Summing those schooling costs for 20 years beginning at age 2 and discounting 3% annually, we estimate the schooling costs of children with severe cognitive impairment to be €747,175.

**Treatment costs for mild hearing impairment (HearingMild)** Hearing loss is not seen in children with CT in the absence of other outcomes. Even though hearing loss might be profound, we estimate the cost only for mild hearing loss (correctable with hearing aid) to avoid double counting for special schooling, parental productivity loss, and other treatment and indirect costs. The average price of a hearing aid in France is based on table 14 in de Kervasdoué and Hartman,^6^ which reports French hearing aid prices from a variety of studies “converging” to €1,535 per unit inclusive of VAT. Assuming bilateral hearing loss, the purchase of hearing aids for both ears would be €3370. The estimated average hearing aid cost assumes the purchase of new hearing aids every 5 years. We assume first purchase of hearing aid at age 2, continuing until age 82 years, the average life expectancy for men and women in France in 2018.^4^ It is further assumed that the annual cost of batteries is €50 and persons with mild hearing loss have a consultation costing €50 every 5 years. Presently, the pace of technological improvements in hearing aids appears rapid, but not wanting to speculate whether that would result in falling prices or improved quality or both, we assumed a constant nominal price. The estimate by de Kervasdoué and Hartmann was in 2016. Adjusting for inflation between 2016 and 2020,^2^ the 2020 cost of hearing aids in France was €3525. Summing quinquennial costs of €3525 over 80 years and discounting 3% annually, we estimate the lifetime costs of hearing aids in France to be €22,343.

**Special education costs for children with severe vision impairment (SpecEdBlind)** is based on the estimate of schooling costs for blind children in Germany in Table 7.9, page 134 of Walter *et al.*^1^ Annual cost in Germany in 2008 was €10,333 euros for 10 years beginning at age 5. Adjusting German schooling costs in 2008 to French consumer prices in 2020, we estimate annual cost of schooling for the blind in France in 2020 to be €11,859.^2^ Summing costs of schooling for blind children for 10 years beginning at age 5 and discounting 3% annually, we estimate the costs of schooling blind children in France for 10 years to be €91,993.

**Treatment costs for mild visual impairment (VisualMild)** Our estimate of the treatment cost of mild visual impairment is based on page 163 in Lafuma *et al*.^7^ They report the yearly average cost for individuals with low vision in France was 7242 euros in 2006, equivalent to 8666 euros in 2020 after adjusting for inflation. Discounting 3% annually, over a lifetime (from age of majority to 82.8 years), we estimate the total cost in France to be 272,684 euros.

**Caregiver earnings loss (ParentEarnLoss)** is based on estimates of caregiver’s work reduction from Tables 1 and 2, page 1131 in Lange *et al.*^8^ as reported in Table 7.9, page 134 of Walter *et al*.^1^ They estimate the caregiver’s annual earnings loss for Germany in 2008 for children younger than 6 years, 6 to 10 years, and 11 to 18 years. We adjust those earnings by the ratio of real average wages in Germany and France in 2008.^5^ The earnings were also adjusted for the difference in consumer prices between Germany and France in 2008 and for consumer price inflation in France between 2008 and 2020. Consumer price levels were measured by changes in the harmonized index of consumer prices.^2^ The caregiver’s lost earnings during each of the child’s first six years was €3,419 or €3526 when adjusted for differences in consumer prices between Germany in 2008 and France in 2020. During each of the subsequent 5 years, the caregiver’s lost wages were €2,084, or €22,150 when adjusted as just described. During each of the last 8 years, the caregiver’s lost earnings were €672, or €693 when adjusted as just described. Summing over 18 years, discounting 3% annually, and adjusting for higher real wages in Germany than in France, we estimate the French caregiver’s earnings loss to be €28,262.

**Severe visual impairment costs and earnings loss (VisualSevere)** Visual severe (non-medical costs) is composed of two separate costs, indirect costs (earnings loss from severe visual impairment) and (direct costs) other non-medical costs associated with severe visual impairment. These costs must be calculated separately since they occur at different periods over a person’s lifetime. Estimates of both costs are based on Table II, page 199 in Lafuma *et al*.^7^ They report estimated annual per person lost earnings from severe visual impairment in both the community and in institutions to be €4002 in France in 2004. The direct cost of severe visual impairment was €12,968 less indirect costs (€4002) = €8,967 in France in 2004. The calculation of lifetime earnings loss from severe visual impairment assumes people without severe visual impairment work from age 18 to the mean retirement age of 62 in France. Life expectancy for men and women in France in 2020 is 82.8 years.^4^ The lifetime direct costs of severe visual impairment in France is €352,347 and the lifetime lost earnings from severe visual impairment in France is €74,124.^2^ Total non-medical costs (including lost earnings) are the sum of the two figures calculated above, the loss of earnings from age 18 to 62 and other non-medical costs incurred over the lifetime, which equaled €590,374.

**Value of a statistical life** (VSL) is routinely used in the economic evaluation of public policies that may lead to higher or lower mortality. As a recent publication of the OECD put it, “The idea of associating a monetary value with human life is very challenging and can seem insensitive or harsh. Life is indeed priceless, at least when considered from the complex perspective of an individual. Policy makers are regularly devising policies and regulations that affect people’s risk of death and that seek to protect lives in society and require methodologies for comparing the costs of reducing risk with the expected benefits in terms of lives saved. The benefits of prevented mortalities can be expressed in terms of a ‘value of a statistical life’ (VSL), which represents the value a given population places *ex ante* on avoiding the death of an unidentified individual” (from the foreword of the executive summary).^9^ VSL is a term that tends to evoke negative reactions among non-economists who say that the value of life should not be expressed in monetary terms. Perhaps a term suggested by Brigitte Desaigues,^10^ “the value of a prevented fatality” would be less objectionable.

The present study is a benefit-cost analysis, that is, it compares the benefits of a maternal toxoplasmosis screening regimen with its costs. An important benefit of the screening regimen is that it reduces the mortality of toxoplasmosis infection. VSL is simply a way to measure the magnitude of the benefit of preventing mortality. In the last several decades, the VSL has been routinely used in benefit-cost analysis of a wide array of public policy choices such as mandating use of automobile air bags, vehicular speed limits, water and air quality standards, and vaccinations.

There is a voluminous literature on measuring VSL in Europe and in industrialized countries generally. Initially, the discourse over VSL often employed a human capital approach to measuring VSL based on the estimated income lost over a lifetime. The human capital approach has been supplanted by the stated preference approach (in which respondents are asked about their willingness to pay for changes in mortality risks) favored by most European economists and the revealed preference approach (which constructs hypothetical markets for mortality risk change and asks survey respondents for their willingness to pay to reduce mortality risk) used by most US economists. The latter two methodologies produce substantially higher measures of VSL than the human capital approach.

The OECD recently assessed over 800 studies in OECD countries that use the stated preference approach to measuring VSL.^9^ That study recommends using an adult VSL ranging between US $1.8–5.4 million (in 2005 US dollars) with median and mean of US $3.6 million. The OECD adds that “when the policy that is analyzed targets children specifically (or affects mainly children), a higher VSL is recommended, based on the available empirical evidence from the United States and Europe. *VSL for children should be 1.5-2.0 times higher than the mean adult VSL*” (page 131, emphasis in original).^9^

Three studies (^10-12^) and the four studies mentioned in OECD ^9^ have measured VSL in France. These studies assign a wide range of estimates to VSL, from $200,000 to $26.5 million.^10,12^ The lack of consensus about the appropriate value of VSL in France suggests a substantial variation in choice of methodology and provides little guidance in the search for a usable measure of central tendency. Rather than base our statistical analysis on seven French studies with widely divergent methods and resulting estimates, we opt to follow the OECD’s recommendation for all of Europe based on hundreds of studies.

We adjust the OECD’s recommendations for VSL measured in US dollars in 2005 to the consumer price level in France in 2020^2^ using exchange rate data from <https://www.statista.com/statistics/412794/euro-to-u-s-dollar-annual-average-exchange-rate/>. The OECD’s recommended adult VSL adjusted to French consumer prices in euros in 2020 are €1.7 million (low), €3.5 million (medium), and €5.3 million (high). After adjusting for inflation and the exchange rate, the median child VSL recommended by OECD is €5,580,837.

At present, most US economists measure VSL using a revealed preference approach based on income differentials among occupations with different mortality risks. Datasets that have become available in the United States allow much more precise measures of income and mortality risk by occupation, industry, gender, age, and other categories. The new data led to measures of VSL that are typically higher than both the earlier measures using the revealed preference approach and current measures using the stated preference approach, which as noted above is commonly used in Europe.^7^ Recent US government benefit-cost analyses use measures of adult VSL that range between US$6 million and US$10 million (in 2013 dollars), equivalent to €4.5‒€7.5 million in 2020 euros. In the United States, however, several authors discourage upward adjustment of VSL for children.^13^ In short, the US and European approaches to VSL, though they use different methodologies and age adjustments, arrive at recommended measures of VSL that are remarkably similar (€2.6‒€7.9 million in Europe vs. €4.5‒€7.5 million in the United States).

**References for Supporting Information: Measuring costs of impairment**

1. Walter E, Brennig C, Schöllbauer V. How to save money: congenital CMV infection and the economy. In: Halwachs-Baumann G, ed. Congenital Cytomegalovirus Infection: Epidemiology, Diagnosis, Therapy. Vienna: Springer; 2011: 121-44.

2. Eurostat. HICP (2015 = 100) - monthly data (index). 2020. <https://appsso.eurostat.ec.europa.eu/nui/submitViewTableAction.do> (accessed July 22, 2020 2020).

3. Gustavsson A, Svensson M, Jacobi F, et al. Cost of disorders of the brain in Europe 2010. *Eur Neuropsychopharmacol* 2011; **21**(10): 718-79.

4. OECD (Organization for European Co-operation and Development). Life expectancy at birth (indicator). 2020. <https://data.oecd.org/healthstat/life-expectancy-at-birth.htm> (accessed 15 July 2020.

5. OECD.Stat (Organisation for Economic Co-operation and Development). Average Annual Wage. 2020. <https://stats.oecd.org/Index.aspx?Data> SetCode=AV AN WAGE] (accessed 15 July 2020).

6. de Kervasdoué J, Hartmann L. Economic Impact of Hearing Loss in France and Developed Countries A survey of academic literature 2005-2015, Final Report 2016. Paris, France: Conservatoire National des Arts et Métiers, 2016.

7. Lafuma A, Brezin A, Lopatriello S, et al. Evaluation of non-medical costs associated with visual impairment in four European countries: France, Italy, Germany and the UK. *Pharmacoeconomics* 2006; **24**(2): 193-205.

8. Lange K, Danne T, Kordonouri O, et al. Diabetesmanifestation im Kindesalter: Alltagsbelastungen und berufliche Entwicklung der Eltern [Diabetes in childhood: everyday burden and professional consequences for parents]. Dtsch Med Wochenschr  2004; **129**(20): 1130-4.

9. OECD (Organization for European Co-operation and Development). Mortality Risk Valuation in Environment, Health and Transport Policies. Paris: OECD Publishing; 2012.

10. Desaigues B, Rabl A, Ami D, et al. Monetary Value of a Life Expectancy Gain due to Reduced Air Pollution: Lessons from a Contingent Valuation in France. *Revue d'économie politique* 2007; **117**(5): 675-98.

11. Herrera-Araujo D, Rochaix L. Does the Value per Statistical Life vary with age or baseline health? Evidence from a compensating wage study in France. *Journal of Environmental Economics and Management* 2020; **103**: 1-18.

12. Desaigues B, Rabl A. Reference Values for Human Life: An Econometric Analysis of a Contingent Valuation in France. In: Schwab Christe NG, Soguel NC, eds. Contingent Valuation, Transport Safety and the Value of Life. Boston. Kluwer Academic Publishers; 1995; 85-112.

13. Dockins C, Maguire K, Simon N, Sullivan M. Value of Statistical Life Analysis and Environmental Policy: A White Paper. U.S. Environmental Protection Agency, National Center for Environmental Economics, Washington D.C., 2004.
