# Supplementary figures and images for: Prevention of congenital toxoplasmosis in France using prenatal screening: A decision-analytic economic model
Source: PLoS One. 2022 Nov 4;17(11):e0273781. doi: 10.1371/journal.pone.0273781 (PMC9635746; doi:10.1371/journal.pone.0273781)

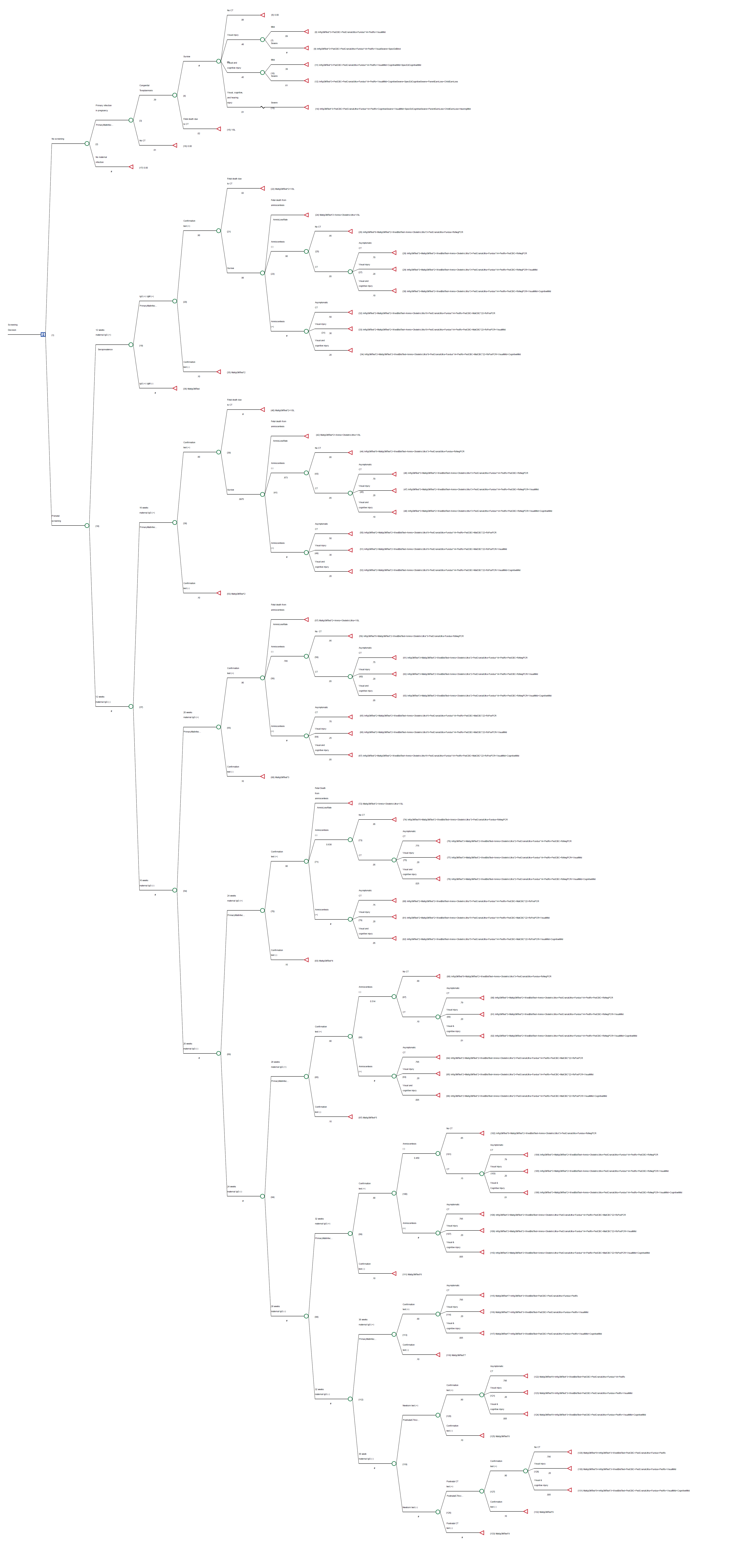

Supplement: S1 Fig — Each outcome has a conditional probability equal to the sum of the probabilities along each branch. The formulas at the terminal nodes for each outcome provide the current cost of testing, surveillance and medications, and costs of all direct injury costs (such as remedial education) and all indirect injury costs (such as lost earnings due to impairment) as shown in S1 Fig. Since the decision tree reproduced into a single image proved to be very difficult to read, we segmented the tree into its 9 main branches (S2–S10 Figs) and a simplified version of decision tree (S11 Fig). (TIF) [file pone.0273781.s002.tif]

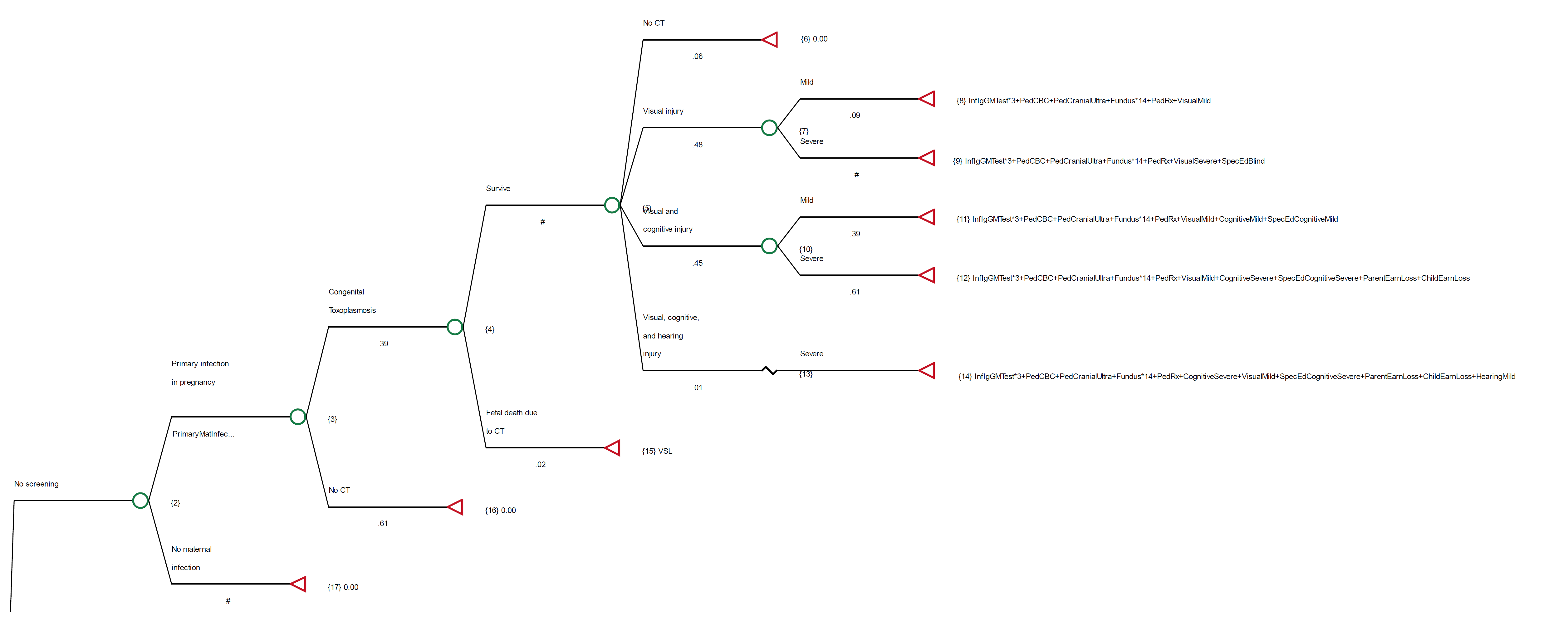

Supplement: S2 Fig — (TIF) [file pone.0273781.s003.tif]

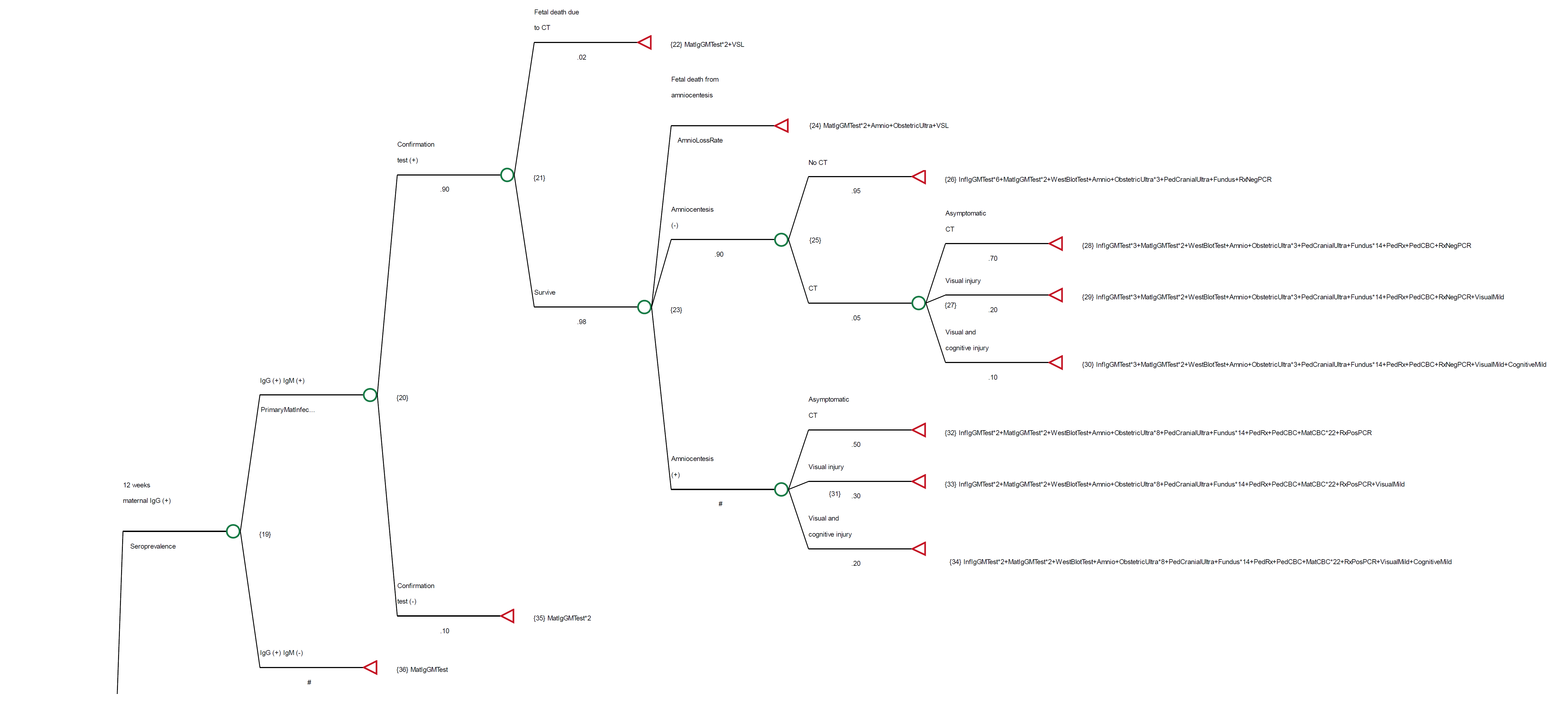

Supplement: S3 Fig — (TIF) [file pone.0273781.s004.tif]

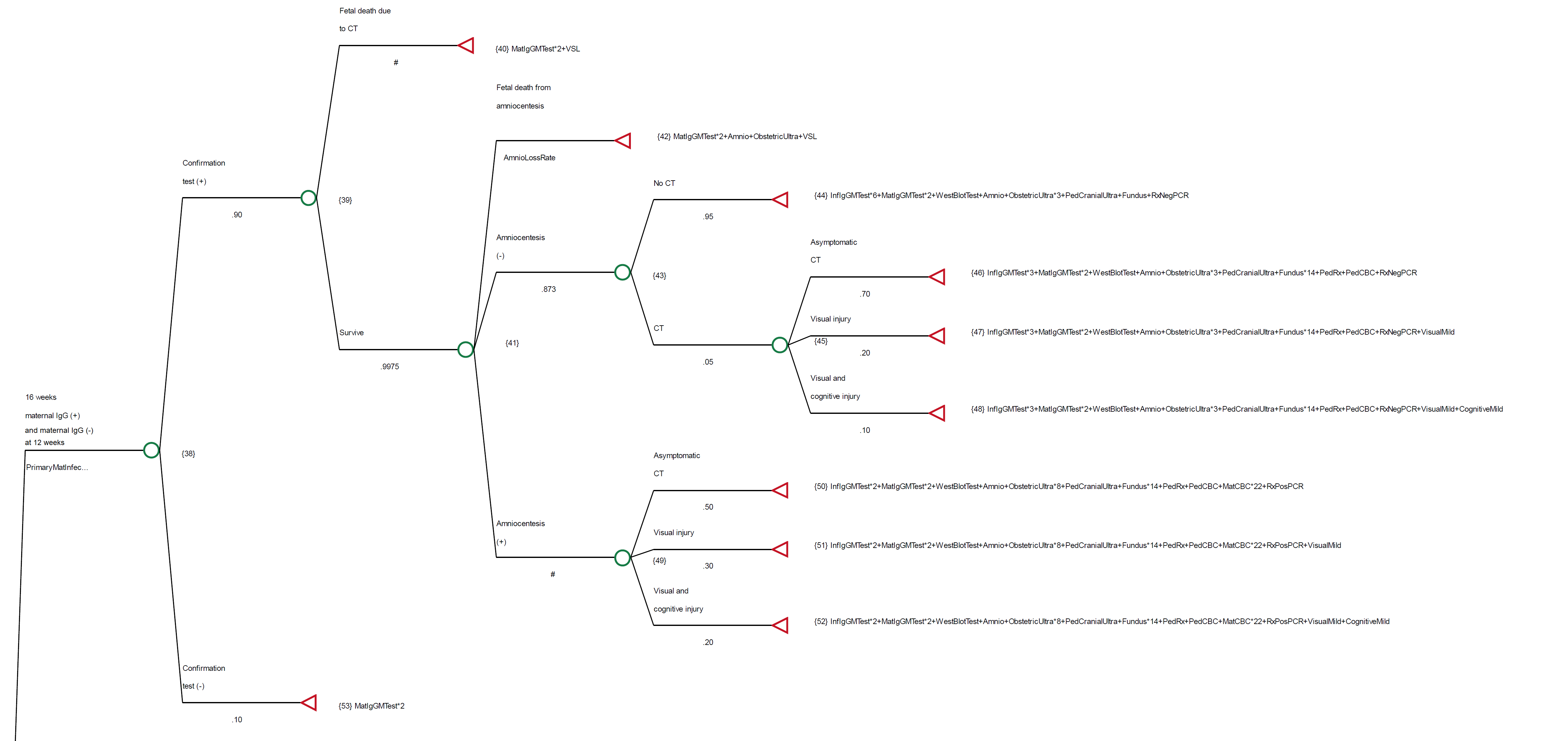

Supplement: S4 Fig — (TIF) [file pone.0273781.s005.tif]

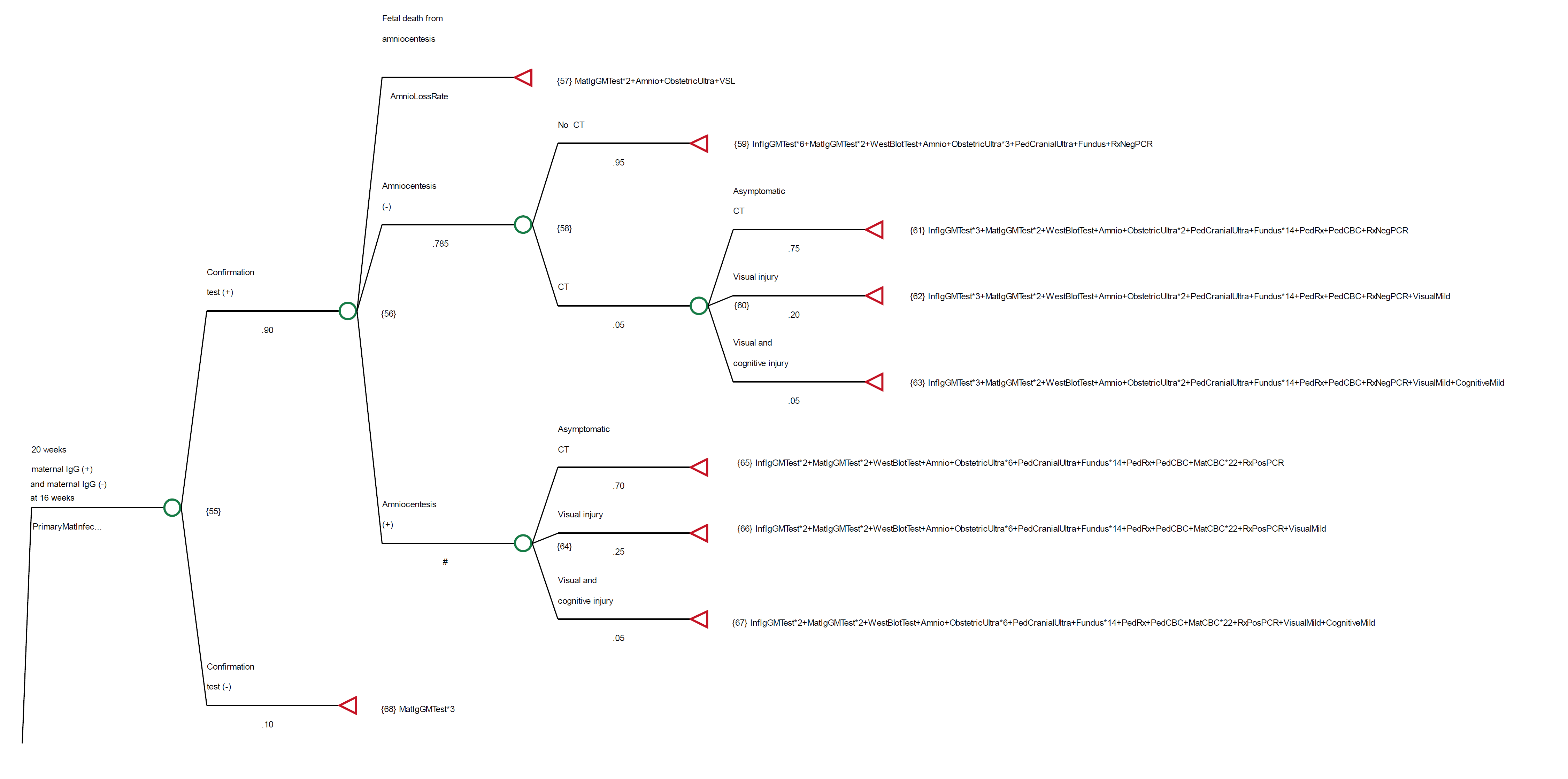

Supplement: S5 Fig — (TIF) [file pone.0273781.s006.tif]

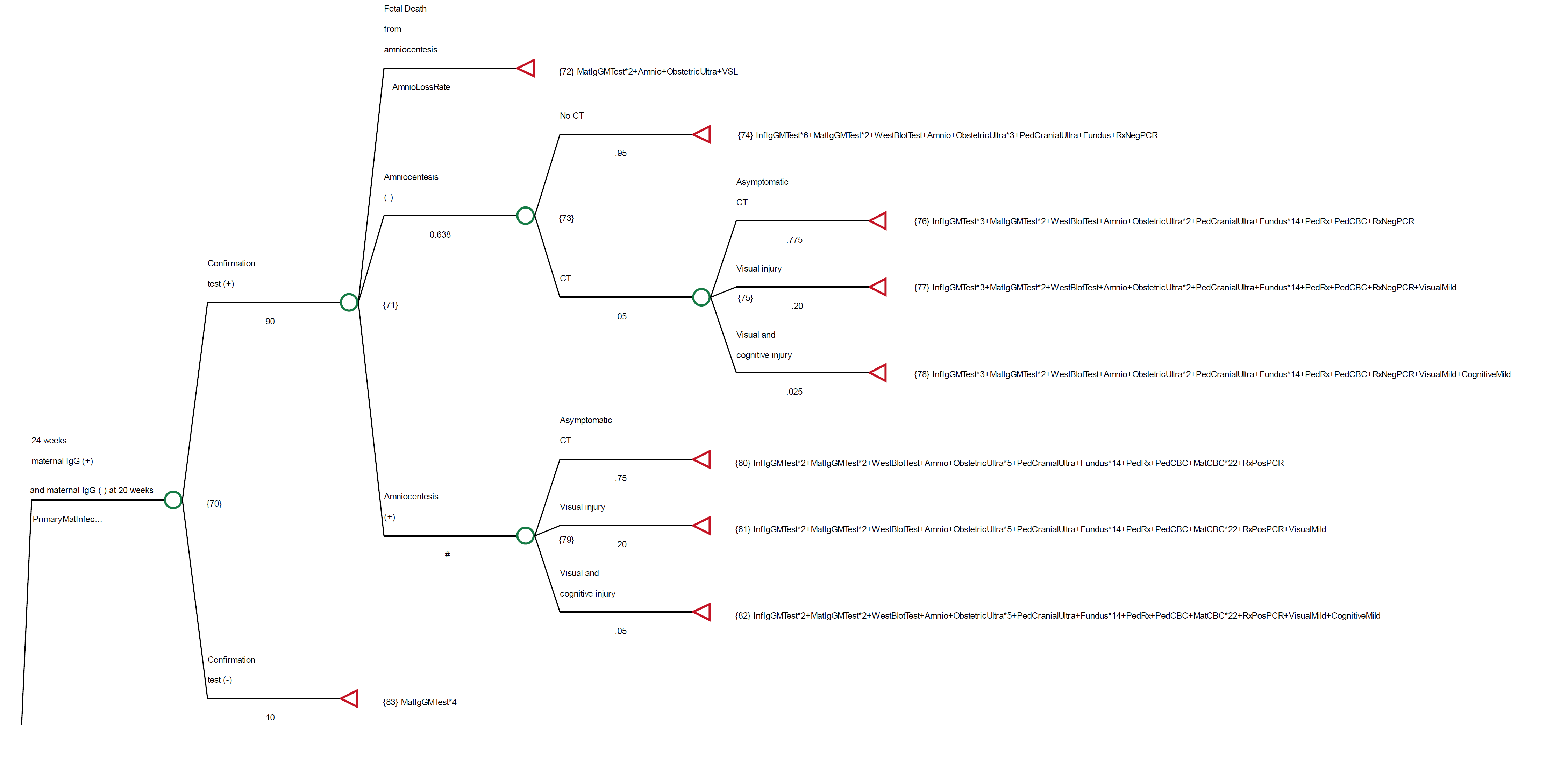

Supplement: S6 Fig — (TIF) [file pone.0273781.s007.tif]

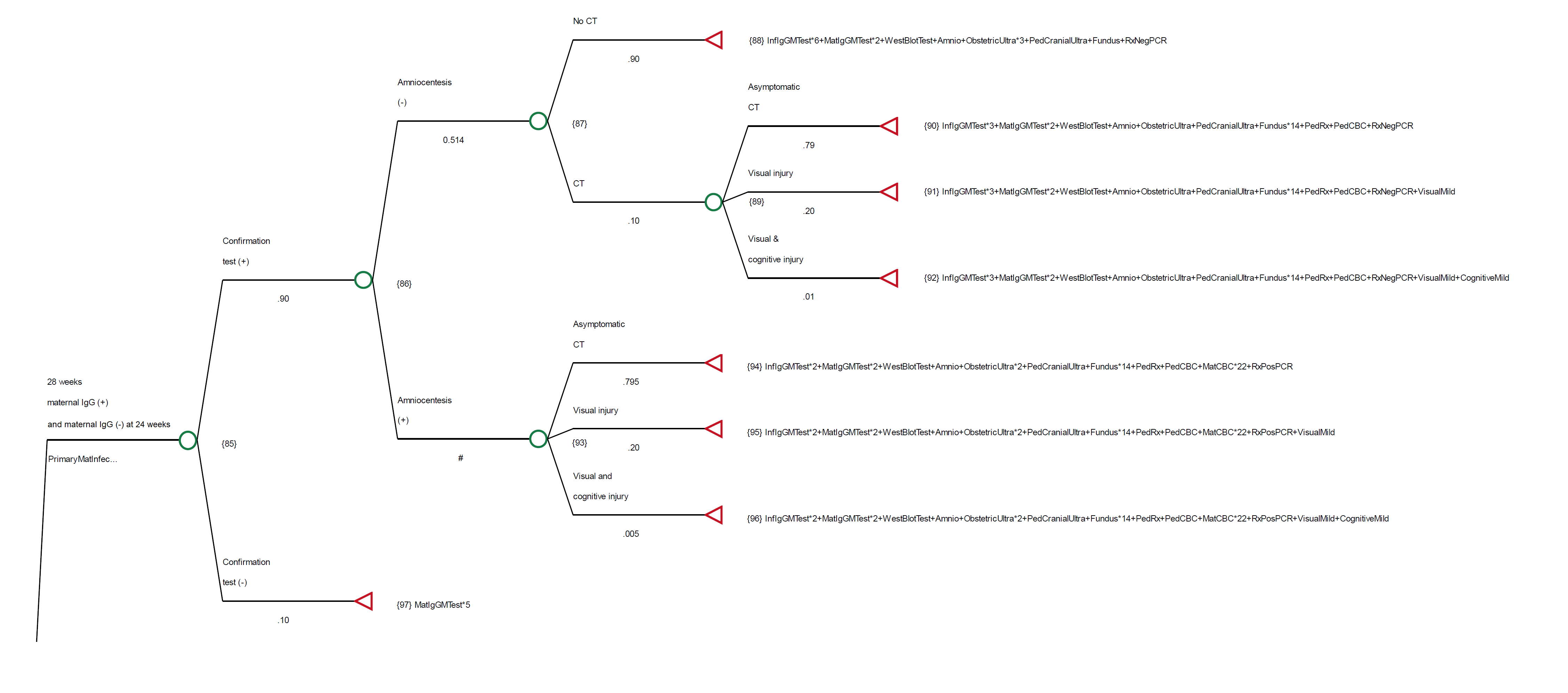

Supplement: S7 Fig — (TIF) [file pone.0273781.s008.tif]

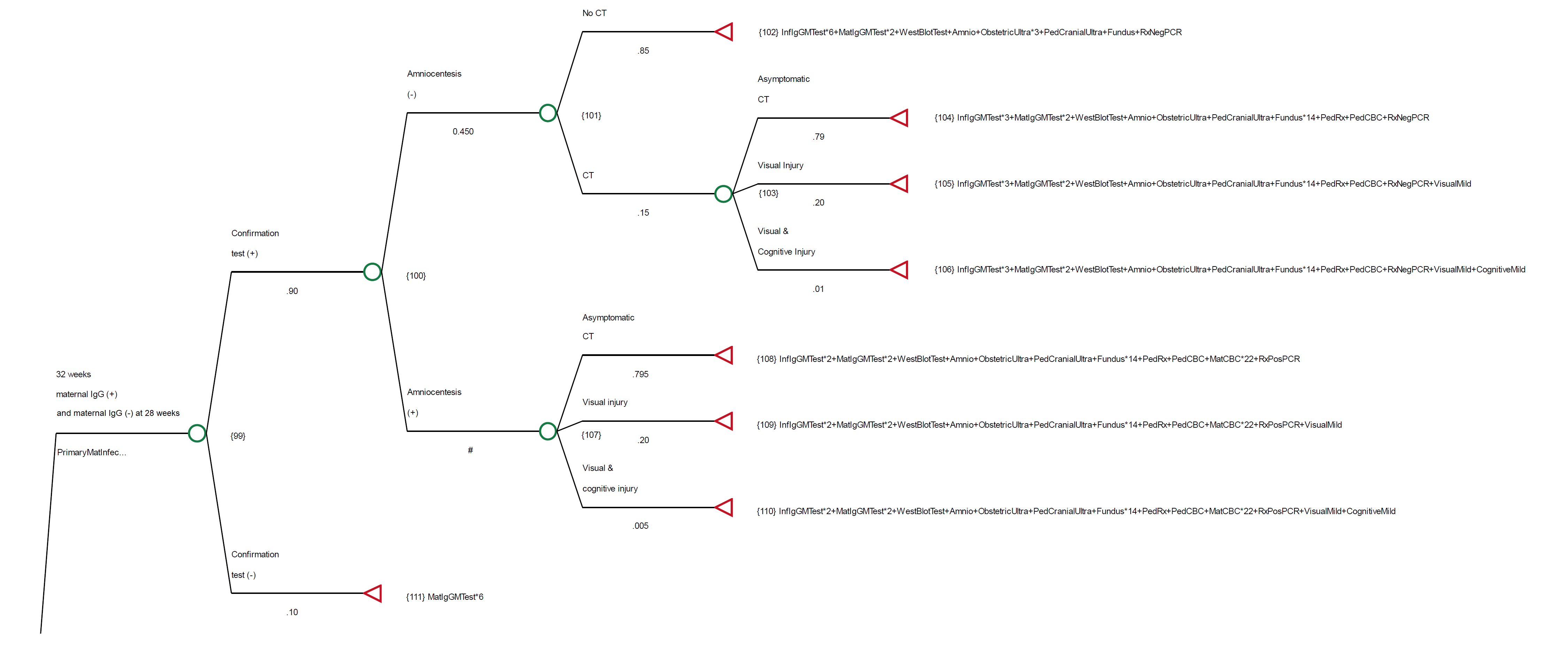

Supplement: S8 Fig — (TIF) [file pone.0273781.s009.tif]

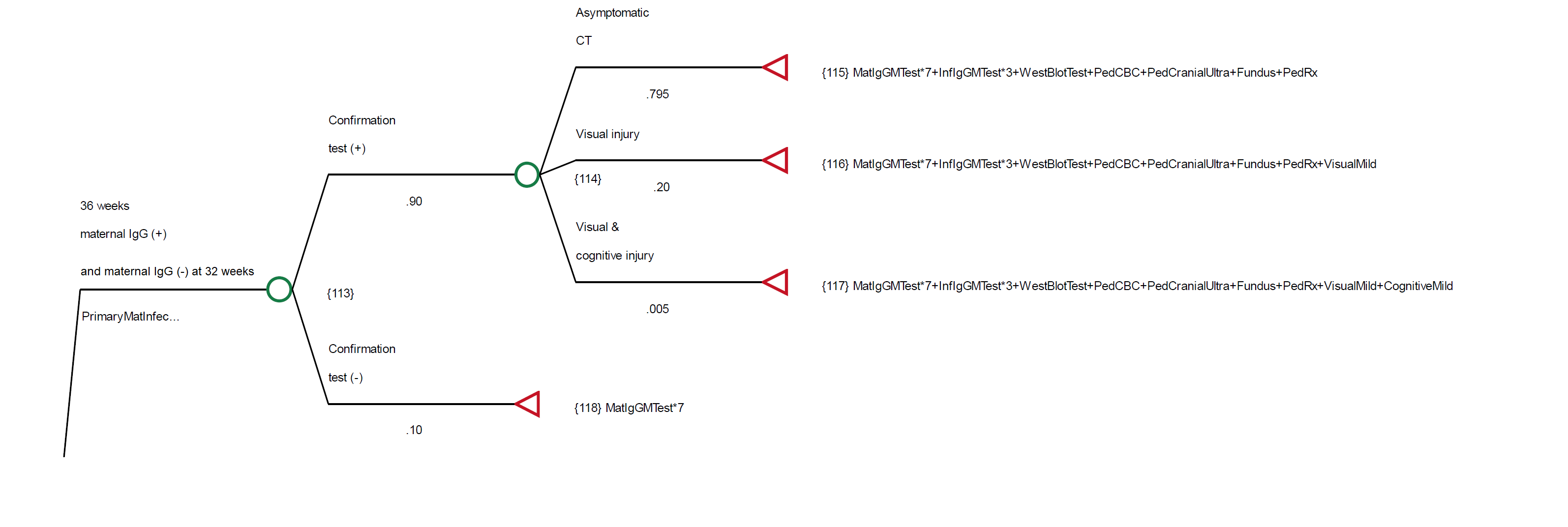

Supplement: S9 Fig — (TIF) [file pone.0273781.s010.tif]

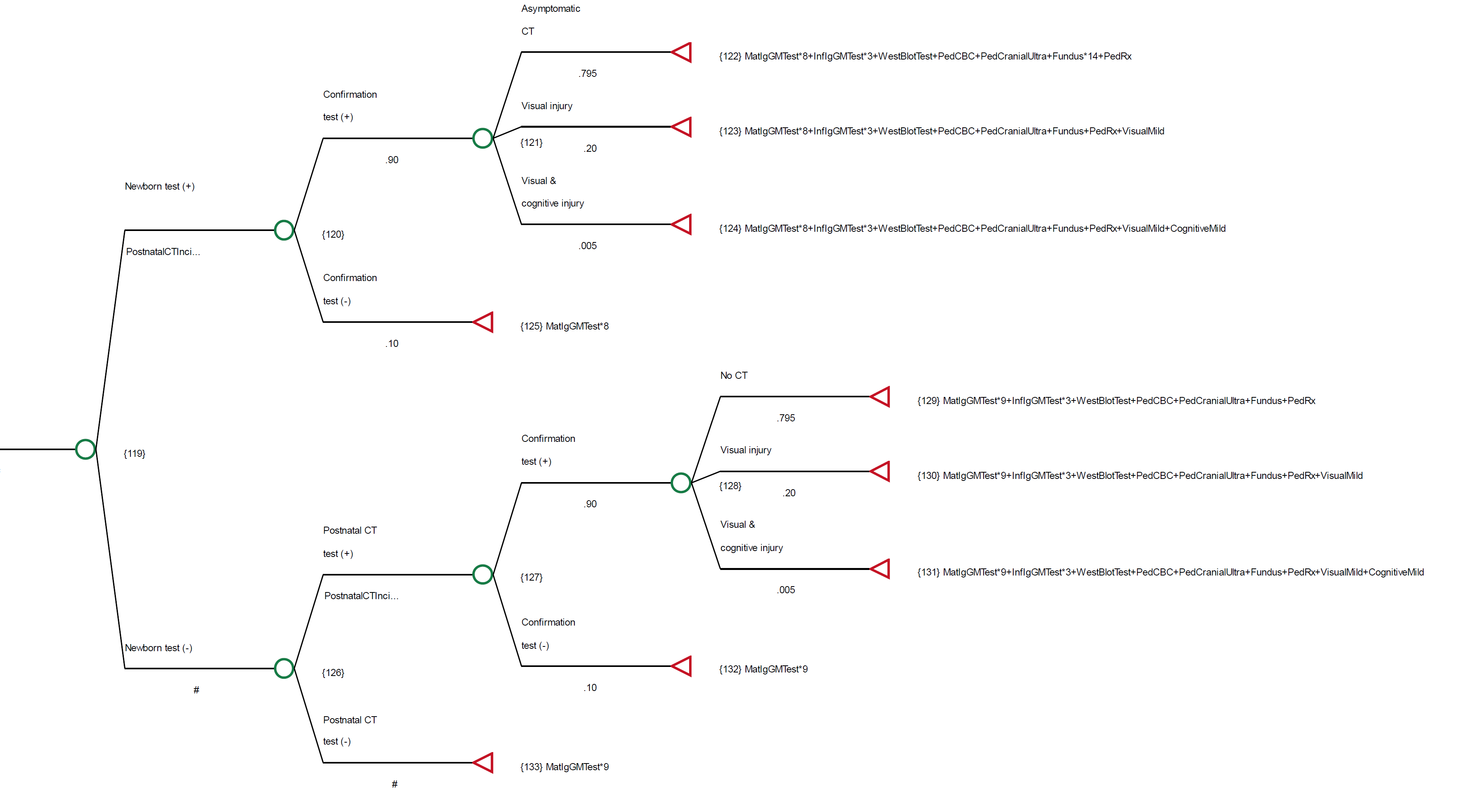

Supplement: S10 Fig — (TIF) [file pone.0273781.s011.tif]

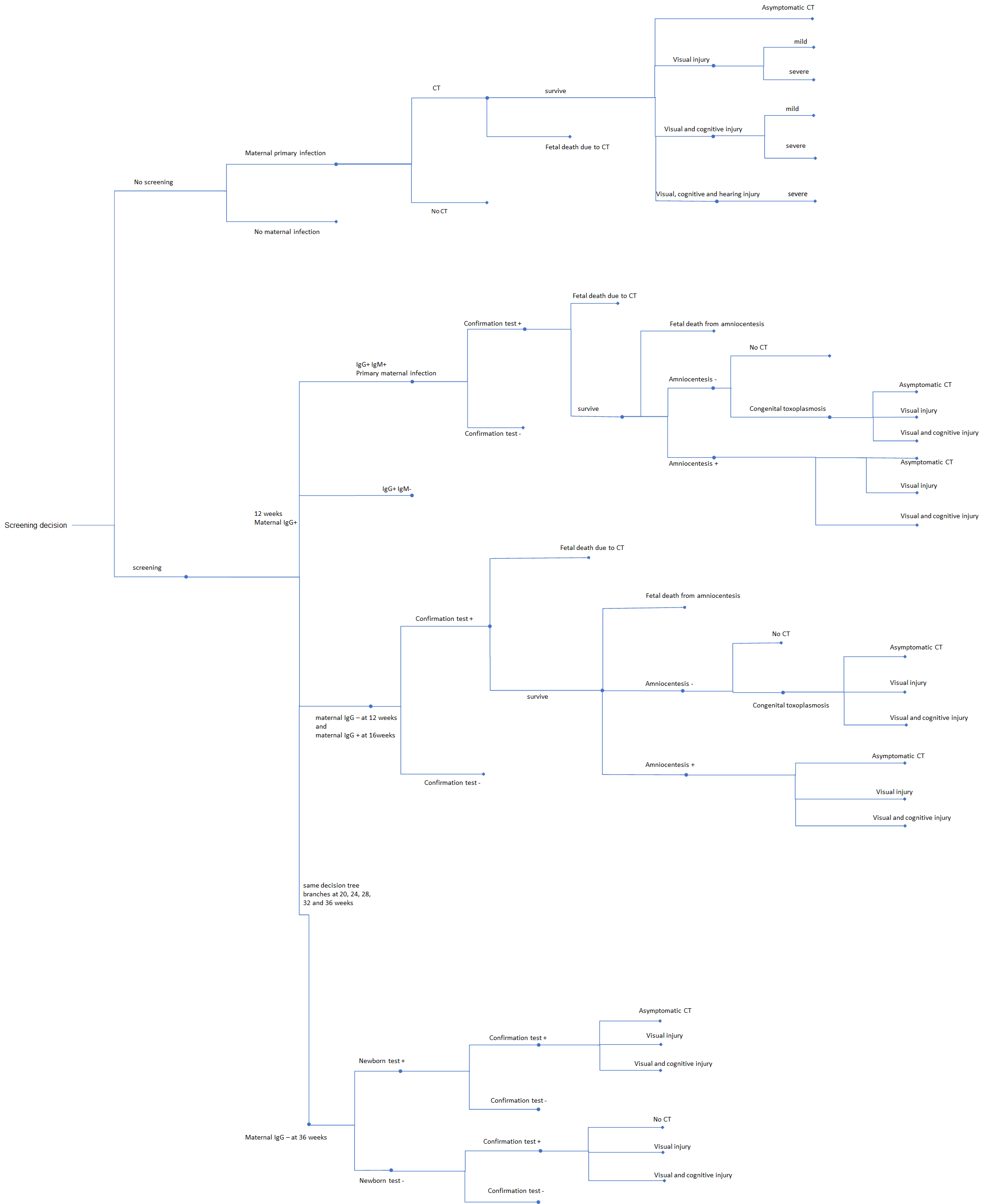

Supplement: S11 Fig — (TIF) [file pone.0273781.s012.tif]
