# Supplementary material for: Prevention of congenital toxoplasmosis in France using prenatal screening: A decision-analytic economic model
Source: PLoS One. 2022 Nov 4;17(11):e0273781. doi: 10.1371/journal.pone.0273781 (PMC9635746; doi:10.1371/journal.pone.0273781)
Supplement: Attachment — Submitted filename: reviewing-09-02-2022.docx [file pone.0273781.s013.docx]

I read with interest the article entitled “Prevention of congenital toxoplasmosis in France using prenatal screening: A decision-analytic economic model”. The goal of this article was to build a decision analysis model to estimate the cost-savings associated with the implementation of a screening program of CT following the protocol described by the National College of French Obstetricians and Gynecologists, and using as often as possible French clinical, epidemiological and economic data.
This is a well written and structured article. A quite important and interesting work has been made concerning the costs estimation to be adapted to the French context.

However, I have some major questions concerning the goal of the article and the methodology. I hope that my comments could also help making this article clearer for the reader.

**Major comments:**

1-When reading the title, I thought that results issued from this study will concern France and their decision makers, but when reading the discussion, it seems that this article is an advocacy in favor of the US screening program, if US were following the French protocol. There is some convergence between results whatever the county considered (France, Austria...) which can be used as a justification to implement screening in the US. But the title of the article doesn’t concern US but France. So, please make clearer the context and the goal of the article and modify the title to remove this important ambiguity.

**2-General method and figures:**

2a-It is not clear at all what has been modelled and what figures 1 and 1a+1b represent. All figures are totally unreadable even zooming on them at +200%. Therefore, it is impossible to understand differences between decision trees. Please find a way to present them more clearly. Please first add a title and if relevant a legend to each figure.

2b-The authors indicate that two models have been built: does Figure 1a represent the Autria screening program and figures 1a+1b the US program following each the French protocol? If there are two models, why is there only one result (€ 656 versus €444)?

**3-Economic method:**

3a-The point of view of the analysis (societal) is missing. Please add it and justify it.

3b-I do not agree with the word “CBA” which is used in the whole text. To my opinion, the analysis you performed consists in comparing costs only (screening *versus* no screening), even if you tried to take into account societal consequences (using VSL and earnings losses). A cost-benefit analysis is not a simple “costs” comparison. Its goal is to compare costs of the screening program to societal benefits which have been monetary valued. The problem is that that the word “cost” is too general and can include two different concepts: “expenses”, and “losses”. But I’m not sure that adding expenses and losses is totally relevant.

In a CBA, the benefits can be estimated using different approaches, mainly the human capital method or contingent evaluation based on preferences methods, what you did in this article. But if a cost-benefit analysis had been really performed, costs and benefits should have been clearly defined and the formula of the social net benefit provided, which is not the case. In fact I think that the TreeAge® software is not adapted to conduct CBA. The software allows calculating for each strategy a simple difference between weighted costs.

So, basically, my comment doesn’t question the costs components of your analysis but the presentation of these components (some of them are costs, others are social benefits and a clear distinction has to be made between both). Please make either adequate changes or clarifications in the whole text to avoid methodological confusions.

4-Line 172: it is not clear what the authors mean by “the economy regarless of who pays”: I assume this is your manner to define the “societal” perspective? Perhaps a more formal definition could be provided? And what do you mean by “economy”? Please reformulate the sentence.

5-The manner how direct and indirect costs have been defined could be discussed in the light of French recommendations. The HAS (French health Authority) now distinguishes between direct and indirect costs based solely on the criterion of production of care. Thus, the time spent by caregivers, and "parent Earnloss" are considered as a (non)medical direct cost because they correspond to the monetary valuation of the time spent helping. The "ChildEarnloss" are considered as an indirect cost because it is a direct consequence for the child in terms of lost productivity. Societal analysis allows you to take into account all of these costs, so this does not call into question your analysis again. Nevertheless, you could more clearly introduce in the cost paragraph the different categories of costs and what they include. For example, I did not understand what you meant by “non-medical cost” in Table 2. Same thing line 40 “It included direct and indirect costs to society of treatment and care, and

the lifetime lost earnings of children and caregivers”.

6-You decided to increase and decrease by 20% all costs. But generally, a one-way sensitivity analysis is performed only on key parameters*, i.e.* parameters which are like ty to vary or to be uncertain. Therefore, a justification of all these variations should be provided.

7-Line 236, you indicated that the monthly retesting is cost saving for French society since it reduces the number of severe cases of CT. But I do not understand this result. Is it an input of the decision tree or an output? Please provide some explanations.

**8-Discussion**

8a-Line 223: I do not understand the start of your discussion about prenatal and neonatal screening whereas you article concern prenatal screening vs neonatal screening.

8b-Line 256, please explain “Another part of the explanation reflects the technological improvements in diagnosis of CT in the decade after 2011”. After the use of PCR what kind of major progress can be noticed?

8c-Line 281: “The first is that probabilities of clinical outcomes were based on population-based

reports”: Are you sure that data are really population-based? Please modify.

8d-Line 286 “Experimental, parasitological, and clinical data indicate that prompt initiation of anti-parasitic treatment following maternal infection reduces the risk of placental transmission.” A reference is missing.

8e-Line 337 “and where the socio-economic level allows access to prenatal diagnosis”: I’m not sure to understand what you mean. A best expression could be “the economic situation of the country”, no? Please reformulate.

**Minor comments**

1-Please systematically add « - » when using words such as cost- effectiveness

2-Line 105: is specificity of PCR on amniotic fluid 100% or close to 100%

3-there is a repetition of several sentences lines 148 – 151 “the order of the variables…. Is equal to 1”.

4-Line 177: a reference should be provided to justify a 3% discounting rate (World Bank and the World Health Organization in the Global Burden of Disease reports?)

5-Line 191: I do not agree with the sentence “ we used a tornado diagram to test the robustness”. A figure can not test uncertainty associated with parameters: it can help representing the results of the one-way sensitivity analyses. We should read some think like “ the robustness of the model was tested using one-way sensitivity analyses”.
