# Supplementary material for: Prevention of congenital toxoplasmosis in France using prenatal screening: A decision-analytic economic model
Source: PLoS One. 2022 Nov 4;17(11):e0273781. doi: 10.1371/journal.pone.0273781 (PMC9635746; doi:10.1371/journal.pone.0273781)
Supplement: Attachment — Submitted filename: Response to Reviewers.docx [file pone.0273781.s015.docx]

**Response to Reviewers**

Dear Dr. Dionne-Odom,

We are now submitting a new version of “*Prevention of congenital toxoplasmosis in France using prenatal screening: A decision-analytic economic model.”*

The latest review process produced two requests for editing. The first was to move the image of the entire decision tree to a Supporting Information folder. We have followed that suggestion.

The second request for change asked us to add a few sentences to our discussion of the assumptions we made in our economic analysis in the methods section of the main manuscript. As requested, we expanded the passage discussing economic costs.

We have also made numerous small edits througout the text. An example is changing “cost benefit analysis” “to cost-benefit analysis.” All changes including these small edits are marked as “tracked changes.”

I hope you will find these changes appropriate.

Larry Sawers
